# Medico-Legal Questions

**Published:** 1907-06-29

**Authors:** 


### MEDICO-LEGAL QUESTIONS.

### PROFESSIONAL ETIQUETTE.

Three cases of considerable importance to the dental and medical professions—all practically involving the same question—have just been decided by the Court of Appeal. We refer to the cases of *Hill* v. *Clifford, Clifford* v. *Timms*, and *Clifford* v. *Phillips*. Messrs. Isidore and Ruby Clifford, being registered dentists, entered into three separate partnerships with Hill, Phillips, and Timms, for carrying on the business of dentists, and the actions related to the right of these three persons to determine the several partnerships on the ground that the Cliffords had, under the provisions of the Dentists Act, 1878, been found guilty of conduct which was "infamous or disgraceful in a professional respect," and had thereupon been removed from the Register of Dentists. The question arose whether the order of the General Medical Council, acting under the powers conferred by the Medical Act, 1858, and the Dentists Act, 1878, was to any and what extent evidence of professional misconduct on the part of the Cliffords. It was contended on behalf of the Cliffords that the order of the Medical Council was *res inter alios acta* and not admissible in evidence to prove that they were guilty of professional misconduct. This view was adopted by Mr. Justice Warrington in the court below; but the Master of the Rolls, in the course of his judgment in the Court of Appeal, said: "I cannot agree with this view. I think the order is admissible as evidence of the existence of conduct which was 'infamous or disgraceful in a professional respect'; the proof of which fact was essential to the validity of the order of the General Council. In my opinion the order of the General Council should be treated on the same footing as an inquisition. Unless and until evidence to the contrary is given, the order suffices to prove that the Cliffords were guilty of statutory misconduct. No evidence was given by the Cliffords to rebut this *prima facie* evidence." Hill's partnership was created by a deed dated October 18, 1905, and it contained a provision that if any partner should at any time during the continuance of the partnership be "guilty of professional misconduct or of any act which is calculated to bring discredit upon or injure the business," or should "become incapable by reason of lunacy or otherwise to take his part in the management, as the case may be, of the business," then either or the other of them might by notice in writing determine the partnership as from the date of such notice. Hill, shortly after the decision of the Council on May 24, 1906, gave notice in writing to determine the partnership, and brought an action for a declaration that the notice was valid and that the partnership was determined, and for consequential relief. "In my opinion," said the Master of the Rolls, "the notice was valid. I think the Cliffords were guilty of 'professional misconduct' within the meaning of the above provision, and it is idle for a man who has been guilty of 'disgraceful conduct in a professional respect,' to contend that he has not been guilty of 'professional misconduct.' I think, further that by reason of the removal of their names from the Register the Cliffords have become incapable of taking part in the management of the business."

The partnership with Phillips was created by a deed dated June 19, 1895. The business was, by clause 2, to be carried on under the style or firm of "Isidore Clifford." By clause 29 it was provided that if any of the partners be "guilty of professional misconduct or of any act which is calculated to bring discredit upon or injure the other of the partners or either of them, or the partnership business, the other partners shall be at liberty to give notice in writing of their intention to determine the partnership hereby created, and thereupon the partnership shall be dissolved and wound up." The Master of the Rolls said: "I think it is quite clear that it was competent to give the notice of dissolution which was given. In my view, all the considerations as to professional misconduct which arose in Hill's case apply to this case. But there is a further peculiarity about this case. Phillips is not a registered dentist, and I infer from a statement made by his counsel in the court below that he is not qualified to be put on the Register. Under these circumstances it seems to me impossible to allow the two Cliffords, who are not on the Register or qualified to be put on the Register, to maintain an action against Phillips, who is also not on the Register, for the purpose of securing the continuation of the professional business of dentists upon the terms of the partnership articles. The whole foundation or substratum of the partnership is gone."

"In Timms's case there was a deed of partnership dated October 2, 1899. Timms gave notice of dissolution, which, for reasons above stated, I think was valid. The result is that in my opinion the judgment appealed against must be reversed. Judgment in Hill's action must be given for the relief asked by the claim, and the two actions brought by the Cliffords against Phillips and Timms must be dismissed."

